# The work of wildfire brigade members in the Amazon: what does ergonomic work analysis reveal?

**DOI:** 10.47626/1679-4435-2025-1539

**Published:** 2025-12-29

**Authors:** Kamila de Almeida Piai, Andréia De Conto Garbin, Fernando Rodovalho, Kelly Polido Kaneshiro Olympio

**Affiliations:** 1 Expossoma e a Saúde do Trabalhador (eXsat) Research Group, Department of Environmental Health, School of Public Health, Universidade de São Paulo (USP), São Paulo, SP, Brazil.; 2 CoRAmazonia Project, Deutsche Gesellschaft für Internationale Zusammenarbeit (GIZ) GmbH, Bonn, Germany.

**Keywords:** occupational health, Amazonian ecosystem, work, wildfires, saúde ocupacional, ecossistema amazônico, trabalho, incêndios florestais

## Abstract

**Introduction:**

Heatwaves and prolonged droughts intensify and expand the occurrence of forest fires. Wildfire brigade members play a crucial frontline role in fire suppression.

**Objectives:**

To understand how Brazilian wildfire brigade members organize their work, identify risks present in real work situations, and propose public health recommendations.

**Methods:**

This qualitative study was conducted using ergonomic work analysis and primary data collection. The analysis included prescribed work, nonsystematic observations of real work activities, and collective and individual interviews with 20 temporary workers hired by federal environmental agencies.

**Results:**

Wildfire brigade members play a central role in fire management, contributing to the preservation of Amazonian biodiversity and sociobiodiversity. Real work demands high physical effort during fire suppression, leading to reports of fatigue, exhaustion, and injuries such as cuts and abrasions. The main contradiction identified concerns access to firefighting areas, which requires constant adjustments and regulations according to the conditions encountered during actual work.

**Conclusions:**

Despite the significant socioenvironmental value and strategic importance of wildfire brigade members in responding to the climate crisis, structural vulnerabilities remain in work organization and social protection. These findings underscore the need for public policies that ensure decent working conditions. Based on the ergonomic analysis, we present public health recommendations applicable to wildfire brigade members across different regions.

## Abstract (pt)

**Introdução:**

Ondas de calor e secas prolongadas intensificam e ampliam a ocorrência de incêndios florestais. Nesse contexto de crise climática, insere-se o trabalho dos brigadistas florestais, responsáveis pela linha de frente no combate ao fogo.

**Objetivos:**

Compreender a organização do trabalho dos brigadistas florestais brasileiros, avaliar os riscos presentes na atividade real e propor recomendações em saúde pública.

**Métodos:**

Estudo qualitativo, baseado na metodologia da análise ergonômica do trabalho, com coleta de dados primários. Foram analisados o trabalho prescrito, observações não sistemáticas das atividades reais e entrevistas coletivas e individuais com 20 trabalhadores temporários contratados pelos órgãos ambientais federais.

**Resultados:**

Os brigadistas florestais desempenham papel central na gestão do fogo, contribuindo para a preservação da biodiversidade e da sociobiodiversidade amazônica. A atividade real exige elevado esforço físico durante o combate aos incêndios, resultando em relatos de fadiga, exaustão e acidentes, como cortes e escoriações. A principal contradição identificada refere-se ao acesso às áreas de combate, que demanda constantes regulações e adaptações diante das condições encontradas no trabalho real.

**Conclusões:**

Apesar do elevado valor socioambiental e da relevância estratégica dos brigadistas no enfrentamento da crise climática, persistem fragilidades estruturais relacionadas à organização do trabalho e à proteção social. Tais aspectos evidenciam a necessidade de políticas públicas que assegurem condições de trabalho decentes. Com base na análise ergonômica realizada, os autores apresentam recomendações em saúde pública aplicáveis a brigadistas florestais de diferentes regiões.

## INTRODUCTION

The global intensification of wildfires has been driven by both climate change and human activity. Prolonged droughts and rising temperatures increase vegetation susceptibility to fire spread. In the United States, the National Interagency Fire Center reported 64,897 wildfire incidents in 2024, which burned approximately 3.6 million hectares. This contrasts with the 56,580 incidents recorded in 2023, responsible for burning around 1.1 million hectares.^1^ In Europe, the European Forest Fire Information System reported that, in 2024, Italy, Romania, Spain, Portugal, France, and Bulgaria were the most affected countries in terms of wildfire occurrence. Portugal, in particular, recorded the largest burned area, equivalent to 1.56% of its national territory.^2^

In Brazil, the situation was equally concerning. In 2024, the National Institute for Space Research identified 278,299 wildfire hotspots, the highest number since 2010. The state of Pará alone accounted for more than 56,000 occurrences, driven by the combination of severe droughts intensified by the El Niño phenomenon and illegal deforestation and agricultural expansion.^3^ These impacts were especially significant in the Amazon, threatening biodiversity and increasing carbon emissions.^4^

Extreme events such as heatwaves and prolonged droughts have been identified by the Intergovernmental Panel on Climate Change as key factors contributing to more intense and frequent wildfires.^5^ According to the Instituto Sociedade, População e Natureza, wildfires threaten both the biodiversity and sociobiodiversity of the Amazon, affecting sustainable production systems of traditional peoples whose livelihoods depend directly on their territories.^6^ Additionally, vegetation burning releases atmospheric pollutants that affect exposed populations, contributing to cardiovascular and respiratory diseases^7^ as well as cancer.^8^

The work of wildfire brigade members is critical. This activity is performed by trained workers play a central role in fire management and in the protection of Amazonian biodiversity and sociobiodiversity. Understanding the socioenvironmental dimensions of this work, as well as its organizational structure and broader context, is therefore essential, particularly in light of the social determinants shaping the health-disease processes to which these workers are exposed.

Accordingly, the aim of this study was to understand how Brazilian wildfire brigade members organize their work, identify risks present in real work situations, and propose public health recommendations. For the purposes of the methodology and results, the findings were organized into two components: the exploratory phase and the phase corresponding to the ergonomic work analysis (EWA).

## METHODS

This study was approved by the Research Ethics Committee of the School of Public Health at the Universidade de São Paulo and by the Brazilian National Research Ethics Commission (CAAE: 74890123.0.0000.5421). All participants were invited in advance, received detailed information about the study, had their questions answered, and signed an informed consent form.

This descriptive qualitative study used primary data collection. The investigation followed the methodological procedures of EWA^9^ and adopted an ethnographic perspective aimed at understanding work through the direct experiences of workers. Collective and individual interviews and nonsystematic field observations were used as data collection techniques.^10^

### STUDY STAGES

#### Exploratory phase

The exploratory phase was conducted between March and April 2024 through field visits performed by the research team. Initial meetings were held with brigade leaders and field managers to gather information on work dynamics and organizational processes. These activities were followed by visits to the bases where wildfire brigade members are stationed along the BR-163 highway (state of Pará).

In total, 14 brigades of the Chico Mendes Institute for Biodiversity Conservation (ICMBio) were visited in two separate work shifts, enabling informal conversations with 32 wildfire brigade members hired by the agency. The team also visited volunteer brigades in Santarém (Pará), including the riverside communities of Maripá and Anã, the Kumaruara Indigenous Territory, and the Alter do Chão Brigade in Belterra (Pará). These visits provided insight into the organization of voluntary work.

The researchers also accompanied an environmental education activity at an early childhood school in the riverside community of Maripá. During this visit, informal conversations were held with 28 additional volunteer and community members, including Indigenous and riverside residents who fight fires occurring near their homes.

To deepen the understanding of wildfire brigade members work activities and the organization of fire suppression efforts in the state of Pará, the team participated in workshops held in the Lower Tapajós region of the Amazon. These workshops were organized by the Institute for Ecological Research (IPÊ), funded by Brazil-Germany Cooperation for Sustainable Development, in partnership with the Ministry of the Environment and Climate Change, ICMBio, the Brazilian Institute of the Environment and Renewable Natural Resources (Ibama)/National System for Wildland Fire Prevention and Suppression (Prevfogo), and civil society representatives, as part of the Federal Volunteer Strategy for Integrated Fire Management.^11^

#### EWA phases

The EWA was conducted between September and December 2024. Initially, documents related to prescribed work were requested for analysis. Based on these materials, the field stage was developed and consisted of three main procedures.

The first procedure involved nonsystematic observation during preparatory activities preceding deployment to a wildfire event. During this open observation, researchers analyzed movement patterns, gaze direction, verbal interactions, body postures, the number of workers involved, and spacing between them. All observations were documented in field diaries.

The second procedure consisted of collective interviews aimed at capturing general verbalizations about real work activities. Initial questions addressed the routine of wildfire suppression, key challenges, perceived risks and difficulties. Participants included wildfire brigade members, squad leaders, and brigade leaders. These interviews lasted an average of 30 minutes, and main points were recorded in field diaries.

Finally, individual interviews were conducted after responses to wildfire events. These interviews followed a semistructured guide with 15 key questions designed to deepen understanding of real work situations and workers’ perceptions of their working conditions and the effects of the activity. Interviews were audiorecorded with informed consent and lasted up to 40 minutes, depending on response depth, with additional field diary documentation.

### STUDY PARTICIPANTS AND SETTING

A total of 20 workers hired by federal environmental agencies, specifically ICMBio and Ibama/Prevfogo, participated in the EWA phase. All held active contracts and had taken part in at least one wildfire suppression event within the 15 days preceding data collection. Among them, ICMBio wildfire brigade members involved in a wildfire in the Nascentes da Serra do Cachimbo Biological Reserve (Altamira, Pará) participated in preparatory activity observations as well as collective and individual interviews. Additionally, Ibama/Prevfogo wildfire brigade members who responded to a wildfire in a federal settlement area in Moju (Pará) and members of a specialized brigade operating in the Jacareacanga Indigenous Territory (Alta Floresta, Mato Grosso) were included in collective and individual interviews.

### DATA ANALYSIS

Data were reorganized whenever overlapping themes emerged, resulting in the identification of two core analytical categories: i) the installation and conditions of brigade bases, including physical infrastructure, ventilation, hygiene, water supply, and sanitation, as well as aspects of food provision and personal care; and ii) work-related dimensions, including hiring and employment relationships, prescribed work, real work, and the meaning attributed to the activity.

Descriptive statistical analysis of participant profiles was conducted using Microsoft Excel.

## RESULTS

### FINDINGS FROM THE EXPLORATORY PHASE

#### The use of fire and the organization of wildfire brigades in Brazil

The National Policy for Comprehensive Health of Rural and Forest Populations (Ordinance No. 2,311/2014) recognizes and values traditional knowledge as an essential component in health promotion and sustainable territorial management.^12^ Among Indigenous peoples of the Amazon, fire has served as a traditional, controlled land-use tool for more than 11,000 years. The testimony below, shared by a community member, illustrates the symbolic and practical understanding of fire’s strength, as well as the deeply rooted knowledge regarding the challenges of controlling it during wildfires:

*“When a mother tells her child, burn the area ahead. No one stops it, no one defeats a young fire.” *Community brigade member, Aningalzinho Indigenous Village, Santarém, Pará, during a discussion circle held at the IPÊ workshop, April 2024.

This account highlights that although traditional peoples possess extensive expertise in cultural fire management, they also recognize the limits imposed by worsening climate change and human pressures, which amplify wildfire occurrence. This perspective underscores the need to integrate scientific knowledge, public policy, and traditional ecological practices to strengthen fire management strategies and support the health of rural and forest populations.

According to the National Integrated Fire Management Policy, fire is classified into three categories. A *wildfire* is defined as any unplanned and uncontrolled fire in forests or other native or nonnative vegetation formations in rural areas that requires suppression action.^13^ Wildfires are further classified by scale as local, regional, or national. The Integrated Fire Management Plan of the Tapajós-Arapiuns Extractive Reserve, developed by ICMBio, regulates the safe use of fire within the territory and issues approximately 2,000 annual permits for *controlled burns *used in swidden agriculture, representing an important prevention strategy in the region.^14^ In addition to *wildfires* and *controlled burns*, *prescribed burning* is also used for conservation purposes and to reduce fuel loads in high-risk areas prior to dry seasons.^13^

**Figure 1:**
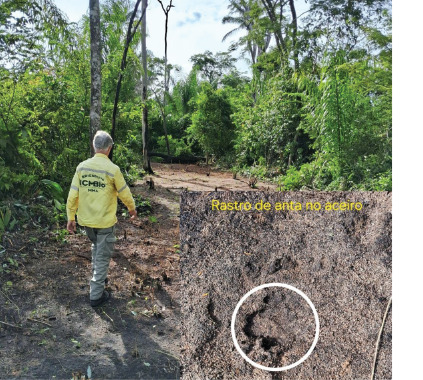
Firebreak technique used to prevent wildfires in high-risk areas of the Nascentes da Serra do Cachimbo Biological Reserve, Altamira (Pará). Source: eXsat archive.

ICMBio guidelines state that fire management in forests and other vegetation formations must include specialized prevention strategies, such as firebreak construction (Figure 1). Firebreaks involve the removal of dry vegetation that serves as potential fuel, creating a cleared strip, manually or mechanically, before critical dry periods to prevent fire spread.^13^

In Brazil, wildfire brigades are composed of workers hired by federal environmental agencies: ICMBio, which oversees protected areas, and Ibama/Prevfogo, which operates in settlements, Indigenous territories, and quilombola communities. Both are coordinated under the Ministry of the Environment and Climate Change. There are also volunteer and community brigades formed by organized local groups acting within their territories.^15^ A survey conducted by IPÊ indicates that volunteer brigades play a significant role, totaling approximately 198 units, which represents nearly 50% of all brigades mapped in the country as of December 2024.^11^

Contracted brigades in the region generally consist of 9-13 members, typically including wildfire brigade members, a squad leader, and a brigade chief. The brigade chief does not remain permanently at a single base but instead rotates among different units. Some bases also include workers responsible for operational and logistical support, who do not engage directly in fire suppression. Volunteer brigades usually include wildfire brigade members and a squad leader, along with members responsible for food, hydration, and communication support. Overall, wildfire brigade members are predominantly men, although women also participate in suppression activities.

The infrastructure of volunteer brigade bases varies widely. These may range from small rooms used for storing equipment, to shared spaces within community associations, to more structured facilities such as those observed in the Alter do Chão brigade. In the case of volunteer and community members, working conditions are particularly precarious, as the absence of formal employment relationships excludes volunteers from the social protection mechanisms established in Articles 6 and 7 of the Brazilian Federal Constitution.^16^

There is an ongoing effort among communities in Santarém (Pará) to strengthen environmental education initiatives. In this context, the activities of the brigade in the riverside community of Maripá were highlighted as a model during the IPÊ workshops held in 2024.^11^ The partnership between the brigade and the local early childhood school has contributed to increasing awareness on wildfire prevention and to training community members who are highly engaged in territorial environmental protection. Based on this exploratory phase, wildfire suppression emerged as the central focus for the subsequent EWA stage.

### RESULTS FROM THE EWA PHASE

#### Demand analysis

In several countries, wildfire suppression is carried out exclusively by professional firefighters. Research focusing specifically on wildland firefighters is relatively recent, and studies on wildfire brigade members remain scarce. Consequently, much of the consolidated evidence on health risks cited in this study derives from the broader firefighting literature, which documents substantial cardiovascular and respiratory strain associated with the occupation.^17^ Furthermore, the International Agency for Research on Cancer classifies firefighting as carcinogenic to humans (Group 1), based on sufficient evidence linking the occupation to mesothelioma and bladder cancer.^18^

Although these findings stem largely from professional firefighter populations, they are comparable to the exposures faced by wildfire brigade members. Particularly notable is continuous exposure to smoke containing toxic substances such as carbon monoxide, resulting from incomplete combustion of organic material, particulate matter capable of carrying polycyclic aromatic hydrocarbons,^19^ and other harmful agents including Cd, Co, Hg, Cr, Mn, Bi, Pb, Sb, and Se.^20^ Such exposures are associated with increased risk of cardiovascular and respiratory diseases, as well as cancer.^7^,^8^,^17^,^18^ The demand addressed in this study was identified based on accounts from supervisors and workers during the exploratory phase, highlighting working conditions and their implications for health.

### PROFILE OF WILDFIRE BRIGADE MEMBERS PARTICIPATING IN THE STUDY

A total of 20 contracted workers took part in the study, with a mean age of 36.3 years. Most participants served as frontline wildfire brigade member (n = 16; 80%), followed by squad leaders (n = 3; 15%) and one brigade chief (n = 1; 5%). All participants had engaged in at least two non-consecutive temporary contracts lasting 6 months each. Of the total, 16 were men (80%) and four were women (20%).

Regarding race/ethnicity, most participants identified as Brown (n = 16; 80%), followed by Black (n = 2; 10%), White (n = 1; 5%), and one Indigenous participant (5%) belonging to the Suruí ethnic group. As for educational attainment, half had completed secondary education (n = 10; 50%); four had incomplete secondary education (20%); four had incomplete elementary education (20%); and two (10%) had completed higher education.

### BRIGADE INSTALLATIONS

Brigade bases are strategically located near forested areas with recurrent wildfire risk. Federal agency bases typically operate on privately owned properties made available for this purpose and generally consist of minimal infrastructure, often small houses on farms. These structures are usually simple wooden buildings equipped with generator-powered electricity and water supplied from artesian wells or natural springs. In many cases, only one bathroom is available with pumped water, and it is common for firefighters to rely on nearby streams for washing.

**Figure 2:**
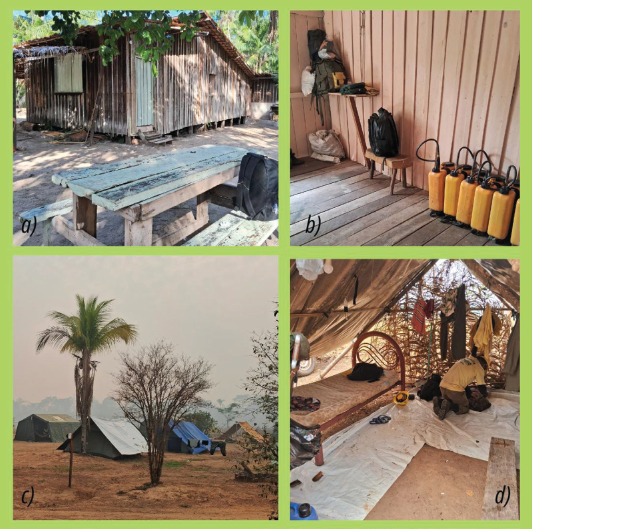
Installations of the brigades participating in the study. Source: eXsat archive.

Note: (a) Base of one participating brigade; (b) indoor equipment storage room, including a 30-liter backpack pump; (c) outdoor area with tents set up during the peak wildfire season; (d) inside view of one of the tents.

Sleeping arrangements typically consist of hammocks set up inside the house. However, during the dry season, when brigades receive reinforcements from other units, indoor space becomes limited, requiring tents to be set up outdoors (Figure 2).

Overall, the interviewed wildfire brigade members consider the infrastructure adequate, though they frequently express the need for more than one bathroom and emphasize water as a critical resource. The presence of a microsystem for pumping and treating water from the well to the base is viewed as a major advantage. Some workers reported bases without nearby springs or wells, complicating daily routines. It is common for federal agency firefighters to rotate between different bases throughout the fire season.

### WORK ASPECTS ANALYZED

Four central aspects of wildfire brigade members work were examined: employment arrangements, prescribed work, real work, and the meaning attributed to the activity.

### EMPLOYMENT ARRANGEMENTS

Wildfire brigade members are hired temporarily and on a large scale during the period preceding the dry season in the northern region of Brazil, generally beginning in June, under Federal Law No. 8,745/1993, which regulates fixed-term contracts established to meet exceptional public-interest needs. These contracts typically last six months and end once wildfire risk decreases. Renewals are uncommon and generally limited to environmental education or reforestation activities. 1- and 2-year contracts may also be issued, with 2 years being the maximum limit for consecutive renewal.^14^,^21^,^22^ Following the enactment of Federal Law No. 15,143/2025, temporary workers of ICMBio and Ibama may now be rehired 3 months after contract termination.^23^

Regarding job functions, ICMBio hires temporary environmental agents and provides specific training in wildfire brigade member. Ibama/Prevfogo hires directly into the roles of wildfire brigade members, squad leader, and brigade chief, which enhances labor protection by recognizing these roles as high-risk occupations and ensuring personal accident insurance and hazard pay.^24^ Salaries range from one to one and a half times the minimum wage (at the time of publication), in addition to legally mandated allowances, including food, transportation, and preschool assistance.

### PRESCRIBED WORK

Federal Law No. 14,994/2024, which established the National Integrated Fire Management Policy, defines the official duties of wildfire brigade members. These include wildfire prevention and suppression; ecosystem and community protection through environmental education and monitoring of highrisk areas; restoration of fire-affected areas; incident record-keeping and documentation; dissemination of information to strengthen institutional cooperation; and participation in continuing education activities.^13^

Contracts establish a 40-hour workweek.^14^,^22^ However, during the critical dry-season months (September to November), wildfire brigade members remain stationed at bases in 15-days-on/15-days-off or 7-days-on/7-days-off rotations, and may remain in the field for more than 45 consecutive days. In these situations, they receive daily stipends.

Contractually prescribed tasks include i) prevention, monitoring, and suppression of wildfires; ii) maintenance and cleaning of facilities; iii) educational activities related to burning practices; iv) seed collection, seedling production, and restoration of degraded areas; v) construction and maintenance of firebreaks, roads, and access routes; vi) support for and execution of prescribed burning; vii) response to emergency mobilizations; viii) physical conditioning exercises; and ix) compliance with internal and safety regulations. Many wildfire brigade members report returning to informal agricultural work after contract termination, primarily in subsistence *roçado,* and note that contract interruptions reduce income and hinder long-term life planning.

The squad leader participates directly in suppression activities, supervises the team, ensures operational safety, oversees equipment maintenance, defines escape routes, and informs the brigade chief of any changes in fire behavior. The brigade chief is responsible for operational and logistical command, alternating between administrative duties and field deployment.^14^ All three roles require specialized training in the prevention, control, and suppression of forest fires, consisting of both theoretical and practical instruction, with a total duration of 40 hours, certified by ICMBio or by Ibama/Prevfogo.

### REAL WORK

#### Wildfire suppression activity

**Figure 3:**
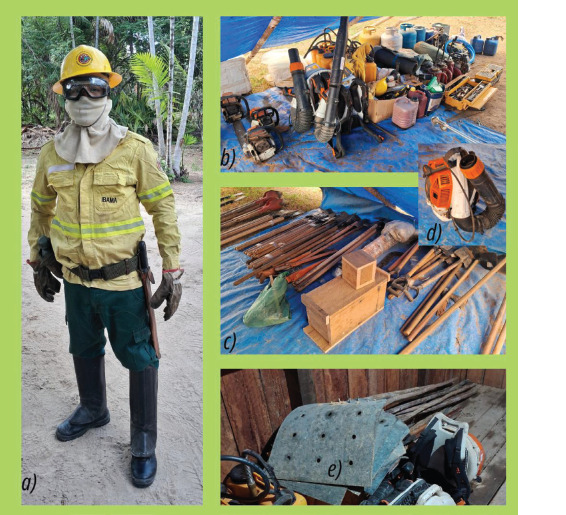
Wildfire brigade member wearing standard PPE, equipment, and tools used during wildfire suppression. Source: eXsat archive.

Note: a) PPE includes a heat-resistant uniform, boots, shin guards, gloves, protective goggles, balaclava, and helmet; also carry a water canteen. b) Equipment transported along forest trails includes a gas cylinder for meal preparation, blower, chainsaw, and rigid backpack pump. c) Tools carried for suppression activities include a sickle, hoe, and machete. d) Blower. e) Fire swatter.

Wildfire brigade members reported using the tools and equipment listed in Table 1, which are carried during the hikes required to access fire sites. These include blowers, rigid backpack pumps, machetes, and hoes, in addition to moving while wearing all their personal protective equipment (PPE), uniform, boots, shin guards, gloves, protective goggles, balaclava, and helmet (Figure 3).

**Table 1:** Tools and equipment used in the field by wildfire brigade members participating in the study

Tools	Type/Purpose
Tools	Type/Purpose
Machete and sickle	Cutting
Hoe	Scraping (removing fuel, uprooting stumps, opening trenches, etc.)
File, handsaw, screwdriver	Auxiliary (tool and equipment maintenance)
Equipment	Type/Purpose
Manual	Rigid backpack pump
Light motorized	Chainsaw, brush cutter, blower
Heavy motorized	Tricycle
Communication	Radio
Navigation/Orientation	Avenza app
Hydration	Water canteen

Wildfire sites are located far from the bases. To reach affected areas, wildfire brigade members rely on combinations of air, river, and land transport. Even when four-wheel-drive vehicles are used up to the entrance of forest access roads, walking distances inside the Amazon Forest may exceed 20 km per day, which already represents a significant amount of work not included in the prescribed duties, increasing fatigue even before suppression activities begin. The following testimony from a squad leader illustrates this demanding effort: *“There was one fire this year where we walked 20 km just to assess an area. And in the last operation I joined, there was a morning when we walked 15 km, carrying pumps, carrying blowers, deep in the forest...*

*we walk a lot! We feel extremely tired. At night, our legs and back hurt, and we get cramps.”*

Squad leader, contracted worker, collective interview, December 2024.

In this way, a series of regulations are enacted to adapt to the situation. During travel through the forest, wildfire brigade members keep their eyes fixed on the ground due to the risk of stumps, holes, and venomous animals. The walk is generally silent because of the high level of concentration required. The neck remains continuously tilted downward to monitor the uneven terrain. Some team members carry oral rehydration solution for potential snakebite incidents.

Frequent minor injuries were reported, particularly small cuts and scratches, often resulting from the use of machetes or sickles. Encounters with bees were also described. Suppression operations are concentrated during daylight hours, as nighttime access increases the risk of accidents involving venomous animals and falls, and visibility remains poor even with headlamps. Mobile phone signal is virtually nonexistent in the forest, but the Avenza app enables offline geospatial navigation.

Hydration is a constant challenge. Because water needs are high and supplies limited, workers often refill their canteens in small forest streams. They frequently camp in the forest to avoid returning to the base and prepare their own meals, which may increase the risk of consuming contaminated water or food, an important concern for public health recommendations directed at this worker population.

## DISCUSSION

Wildfire brigade members in Brazil is carried out predominantly under temporary contracts, which restricts access to labor rights, social security, and formal mechanisms of social protection. These conditions reinforce the socioeconomic vulnerability of workers and reflect a broader process of structural precarization, characterized by the absence of guarantees such as retirement benefits, paid leave, supplemental medical assistance, and minimal stability necessary for long-term life planning.^25^ The situation is even more fragile among volunteer and community workers, who lack formal employment relationships and are therefore excluded from the rights established in Articles 6 and 7 of the Brazilian Federal Constitution.^16^

In this context, legal frameworks that promote stable employment relationships and expand social protection for wildfire brigade members are essential and align with the recommendations of the International Labour Organization (ILO)^26^ and with Sustainable Development Goal 8 (SDG 8), which promotes decent work and inclusive economic growth.^27^

Professional regulation for this occupational group is currently under discussion in the Brazilian Congress through Bill No. 3,621/2024, under review since November 2024 (at the time of this publication).^28^ The importance of this legislative process was reinforced by the motion of support issued by the National Environmental Council in February 2025, which emphasized the need for regulation to strengthen legal security and professional recognition.^29^

The main contradiction identified in wildfire suppression work relates to access to fire-affected areas, a recurrent barrier to task execution. Workers adopt self-protective strategies rooted in practical experience, such as prioritizing daytime operations, moving in teams, and using full protective gear, especially given the risk of encounters with venomous animals. Collective regulation is constant: workers maintain visual proximity to one another and never travel alone, always moving in pairs or groups. This mode of organization demonstrates how real work depends on cooperation as a fundamental safety resource and as a strategy to manage variability. However, this dynamic imposes postural costs, such as the need to maintain the neck flexed for long periods, and cognitive costs, including continuous attention to uneven terrain.^30^

Variability is also expressed across biomes: in the Cerrado, fire swatters are the predominant tool, whereas in the Amazon, blowers are used more frequently. This environmental adaptation illustrates how real work adjusts to local conditions, but it also generates significant ergonomic costs, including carrying heavy equipment, working in sustained bent postures, and performing prolonged physical exertion, factors that contribute to musculoskeletal risks and fatigue.

The distribution of responsibilities regarding tools and equipment follows practical criteria based on accumulated experience rather than strict adherence to formal prescriptions. This reflects the presence of a *collective métier,^30^* in which shared know-how guides critical decisions in the field. A notable example involves the use of blowers and chainsaws: workers who operate these tools often refuel them with gasoline and two-stroke oil without wearing all required PPE. This reveals a contradiction between prescribed work (full protective equipment) and real work (risky adjustments made to avoid interrupting the task).^9^,^30^

Wildfire brigade members carries strong symbolic meaning, frequently associated with protecting the Amazon, safeguarding local communities, and preserving subsistence. The significance attributed to the activity stems both from social recognition and from the sense of belonging and pride reported by workers, which functions as a powerful source of motivation. Nonetheless, a striking contradiction persists: despite the symbolic value and heroic image associated with the role, the work is performed under conditions marked by risk, instability, and material vulnerability. This paradox illustrates the disconnect between the social value attributed to the profession and the human costs involved.^9^

Analysis of real work highlights i) contradictions between prescribed and real work; ii) high variability due to environmental conditions and available resources; iii) individual and collective regulations that ensure continuity of activity; and iv) significant health risks, including prolonged exposure to high temperatures, extreme physical demands associated with dehydration, which may lead to renal strain, along with eye irritation and respiratory symptoms such as dry cough, rhinorrhea, and sneezing. These findings underscore the importance of understanding real work as a basis for improving working conditions and ensuring health, safety, and dignity for wildfire brigade members.^9^,^30^

### PUBLIC HEALTH RECOMMENDATIONS

The public health recommendations presented below were developed based on the EWA findings from the study sample; however, they may be applied to wildfire brigade members across the country:

1) Professional regulation, as a starting point for ensuring labor protections and recognizing the value of this activity, thereby contributing to the promotion of decent work.
2) Creation of a specific code in the National Medical Care System to register health problems related to wildfire brigade members, an urgent measure to improve nationwide monitoring of occupational health outcomes in this group.
3) Updated vaccination protocols aligned with occupational risks, including immunization against typhoid fever, hepatitis A, tetanus, and hepatitis B, in addition to routine immunization campaigns such as influenza and COVID-19.
4) Systematic guidance on safe water and food disinfection during camp operations in remote areas, particularly during prolonged suppression activities.
5) Reinforcement of full PPE use in all activities whenever collective protection measures have been exhausted.
6) Eye cleansing with saline solution or clean water after smoke exposure, preceded by proper hand hygiene.
7) Guidance on appropriate uniform changing practices to avoid cross-contamination with chemical residues.

### FINAL CONSIDERATIONS

The public health recommendations identified in this study are applicable to wildfire brigade members in all Brazilian states, as they represent general occupational health guidelines regardless of the territory where these workers operate. The predominantly temporary employment arrangements reinforce the contractual fragility inherent in this work activity. As discussed throughout this article, the socioeconomic vulnerability experienced by firefighters contradicts the principles of SDG 8, which seeks to promote decent work and sustainable economic growth as outlined in the United Nations 2030 Agenda.

The absence of employment stability and of public policies that recognize and value this workforce, including formal career recognition, may negatively affect both the physical and mental health of wildfire brigade members, ultimately compromising the effectiveness of fire management in their territories. Although these workers are widely regarded by the public as heroes, symbolic recognition does not substitute for institutional and material acknowledgment. The lack of professional regulation prevents the establishment of stable labor guarantees, revealing a paradox between the high socioenvironmental importance of their work and the limited concrete valuation of the category, which contradicts the principles of dignity at work and social protection established by ILO.
